# Corollary Discharge Failure in an Oculomotor Task Is Related to Delusional Ideation in Healthy Individuals

**DOI:** 10.1371/journal.pone.0134483

**Published:** 2015-08-25

**Authors:** Raphaëlle Malassis, Antoine Del Cul, Thérèse Collins

**Affiliations:** 1 Laboratoire Psychologie de la Perception, Université Paris Descartes & CNRS, Paris, France; 2 Service de Psychiatrie d'Adultes, Groupe Hospitalier Pitié Salpêtrière, ICM SAN TEAM, Université Pierre & Marie Curie, Paris, France; University of Ulm, GERMANY

## Abstract

Predicting the sensory consequences of saccadic eye movements likely plays a crucial role in planning sequences of saccades and in maintaining visual stability despite saccade-caused retinal displacements. Deficits in predictive activity, such as that afforded by a corollary discharge signal, have been reported in patients with schizophrenia, and may lead to the emergence of positive symptoms, in particular delusions of control and auditory hallucinations. We examined whether a measure of delusional thinking in the general, non-clinical population correlated with measures of predictive activity in two oculomotor tasks. The double-step task measured predictive activity in motor control, and the in-flight displacement task measured predictive activity in trans-saccadic visual perception. Forty-one healthy adults performed both tasks and completed a questionnaire to assess delusional thinking. The quantitative measure of predictive activity we obtained correlated with the tendency towards delusional ideation, but only for the motor task, and not the perceptual task: Individuals with higher levels of delusional thinking showed less self-movement information use in the motor task. Variation of the degree of self-generated movement knowledge as a function of the prevalence of delusional ideation in the normal population strongly supports the idea that corollary discharge deficits measured in schizophrenic patients in previous researches are not due to neuroleptic medication. We also propose that this difference in results between the perceptual and the motor tasks may point to a dissociation between corollary discharge for perception and corollary discharge for action.

## Introduction

Identifying the sources of our sensations is fundamental to normal cognition. Every body movement–arms or eyes–leads to sensory changes that are due to the displacement of the effector, and not to the outside world. An adaptive system should differentiate between the internal versus external causes of sensory changes. To understand how this differentiation is achieved by the nervous system, the example of the oculomotor system is particularly telling: every eye movement displaces the retinal location of visual objects, yet we do not experience the visual world as constantly shifting. On the contrary, we have the impression of a stable visual world.

Prediction plays a key role in differentiating internal from external sources [1,2]. A copy of the outgoing motor command, called corollary discharge, can generate a prediction about the expected sensory outcome of the movement. This prediction can be compared with the actual sensory reafference to determine whether the sensation is due to the movement or to the outside world [3]. In the oculomotor system, corollary discharge carries information about saccade metrics and can serve to predict the expected retinal consequences of that saccade.

Failure to identify oneself as the source of one’s own sensations (i.e. misattribution of agency) occurs in schizophrenic patients presenting positive symptoms such as auditory hallucinations, thought insertions or delusions of influence [4], in which patients experience their own speech, thought or actions as being caused by an external agent rather than by their own will.It has been proposed that these symptoms could be underscored by low-level deficits in sensory-predictive mechanisms [5,6]. Indeed, previous research with schizophrenic patients has revealed deficits in predicting the sensory consequences of self-generated actions, in the auditory [7,8], sensorimotor [9–12], and visual [13–15] modalities. Moreover, clinical scores of delusions of influence correlate negatively with the ability to cancel out the sensory consequences of self-generated movements [13,16].

A complementary approach to exploring the link between agency disorders in schizophrenia and sensory-predictive mechanisms is to quantify delusion-like thinking in healthy individuals, and test whether this correlates with sensory-predictive measures. Using this approach, Teufel et al. [17] found that the degree of tactile sensory attenuation arising from predictive processes correlates negatively with the tendency towards delusional ideation. Such an approach allows researchers to test mechanistic models of symptoms without the confounding factors of medication, clinical history, and other cognitive impairments often associated with schizophrenia.

The present study examined sensory predictive mechanisms in two visuo-oculomotor tasks in young healthy adults, and correlated performance with a measure of delusional-like ideation in healthy subjects [18]. The double-step task measures the use of a corollary discharge for action, and the in-flight displacement task measure corollary discharge for visual perception. Because good performance in these tasks requires accurate metric knowledge about saccades, we hypothesize that performance will correlate negatively with the tendency towards delusional ideation. Additionally, measuring behaviour from the same subjects in both a motor and a perceptual task allowed us to test the hypothesis of a dissociation between self-movement information for action and for perception, and the respective relationship to delusional ideation.

## Methods and Materials

### Subjects

Forty-one healthy adult subjects (24±5 years old, 21 women) participated in the experiment. Subjects were recruited from the laboratory subject pool and were naïve as to the goal of the study. None reported any current or past psychiatric diagnoses or visuo-motor impairments. All had normal or corrected-to-normal vision, gave their written informed consent prior to starting the experiment, which was carried out according to the ethical standards of the Declaration of Helsinki (2004), and received payment of 10€/hour. The study received approval from the local ethics committee (Comité d’Evaluation Ethique en Recherche Biomedicale, Université Paris Descartes).

### Tasks

Subjects performed two tasks in counterbalanced order.

**Fig 1:**
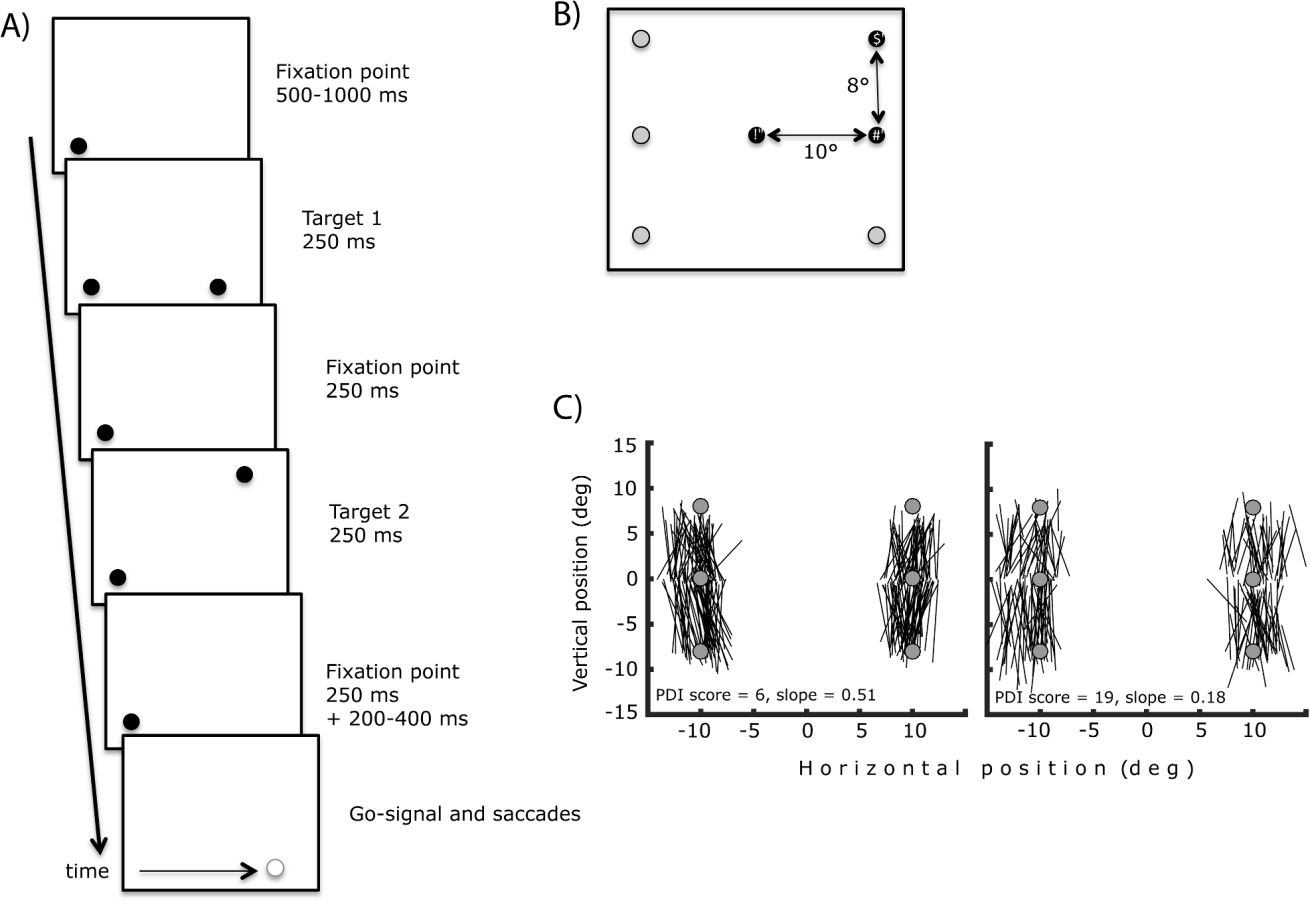
(A) Double-step task. After a 500–1000 ms fixation, the first target appeared for 250 ms, followed by a 250 ms blank (fixation target still on), the second target for 250 ms, another 250 ms blank. After an additional 200–400 ms, the fixation point turned off, indicating to the subjects that they were to perform their saccades. In the visually-guided version (not illustrated), the targets appeared successively and remained on until the end of the trial. (B) Possible target location combinations. First saccade targets appeared 10° to the left or right of the fixation point, second saccade targets above or below the first target. There were thus four patterns: left-up, left-down, right-up, right-down. The example illustrated the right-up pattern. (C) Sample traces from 2 subjects. Each line represents a single second saccade. For each subject, PDI score and the slope from regression between the first saccade error and second saccade horizontal component (see Results section).

The first visuo-motor task was the double-step task [19], in which two visual targets are flashed successively on a screen (Fig 1A and 1B). Subjects are asked to perform memory-guided saccades to the remembered locations of the targets. Performing the first saccade is relatively easy, and requires only transforming the visual information about target location into a motor vector appropriate for guiding a saccade. If the second saccade applied the same sensory-motor transformation, however, the second saccade would grossly miss the (remembered) target location because the eyes have moved since the visual information was encoded. Correct second saccades therefore require information about the first saccade to be combined with the remembered second target location. The fact that humans and macaque monkeys perform the double-step task with ease is evidence in favor of a self-movement, or corollary discharge-like signal (CD) being used in this task. It is also possible to obtain a quantitative measure of CD information use in this task, by examining the relationship between first and second saccade errors [20–22]. Indeed, if the saccadic system possesses specific metric information about the first saccade, then the size of the second saccade should correlate with the first saccade because it would correct for targeting errors occurring on the first saccade. If no CD information contributes to the second saccade, then first and second saccade amplitudes will be independent. The correlation coefficient is therefore an index of CD information use in the double-step task.

Subjects also performed a control version of the task, in which the two flashed targets reappeared at the moment the fixation point disappeared. The targets were thus present while the subject performed the sequence of saccades. This visually-guided task served to check that subjects were capable of remembering target order and performing two successive saccades.

**Fig 2:**
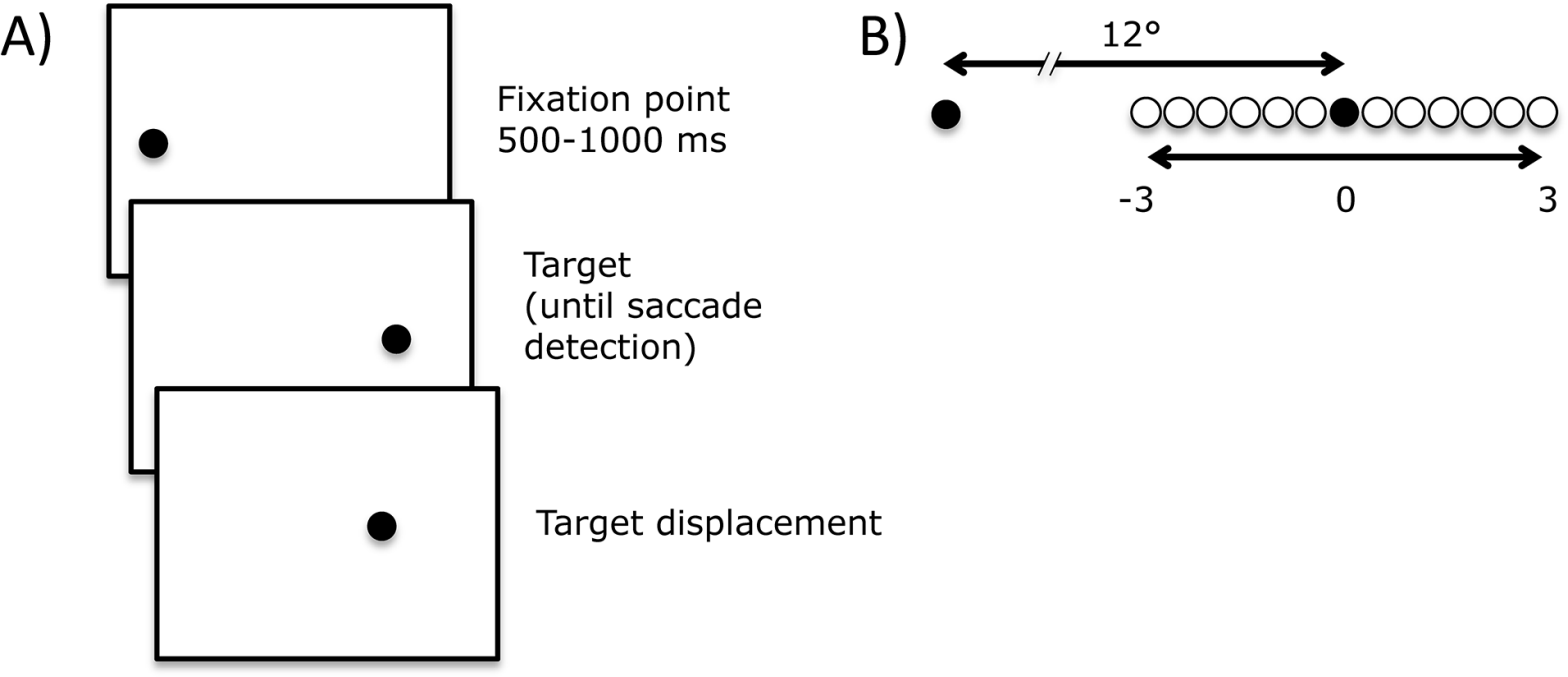
(A) In-flight displacement task. After a 500–1000 ms fixation, the first target appeared and the fixation point turned off. Upon saccade detection, the target was displaced, and subjects reported the direction of displacement. (B) 13 possible equiprobable displacement locations, spanning -3 to 3° from the initial target location in steps of 0.5°.

The second task was the in-flight displacement task (Fig 2A and 2B). Subjects performed a saccade to a single peripheral target that was displaced when the saccade was in mid-flight. Upon landing, subjects were to indicate whether the target had been displaced to the left or to the right by pressing the appropriate button. In other words, they had to perform a trans-saccadic location judgment. What information is available for this trans-saccadic comparison? The most salient post-saccadic visual information is the current retinal location of the target. One simple strategy to solve the task would be to respond based on this retinal information: respond “forwards” if the post-saccadic target is beyond the new foveal location, respond “backwards” if it is behind. This would have the unfortunate consequence of yoking the perceptual experience of target location to saccade accuracy: undershooting saccades would lead to “forwards” responses, overshooting saccades to “backwards” responses. The perceptual responses of healthy adult participants do not depend on saccadic landing positions [23–25]. Some other, extra-retinal, information is thus being used. The obvious candidate is a CD signal generating a prediction of the expected post-saccadic target location, which is then compared to the actual post-saccadic target location. Thus, an undershooting saccade leads to the expectation of a non-foveal target upon landing. The relationship between landing positions and perceptual responses is thus an index of the degree to which the extra-retinal information is available.

Subjects performed three sessions in counterbalanced order: the visually-guided double-step task, the memory-guided double-step task, and the in-flight displacement task. The double-step tasks were composed of 216 trials each, 54 for each of the four saccade patterns. The in-flight displacement task was composed of 468 trials, 36 for each of the 13 target displacement values.

### Stimuli and instruments

Stimuli were 0.5° black dots presented on a 22” FormacProNitron 22800 screen with a resolution of 1024x768 and a refresh rate of 100 Hz. The background was grey, and the room was dimly lit. Participants were seated 57cm from the screen and their head kept stable by chin- and forehead-rests. In the double-step tasks, the fixation point appeared near screen center (the absolute position was selected randomly from four possible locations: 1° to the left or right; 1° above or below screen center). The first target appeared 10° to the left or right of the fixation dot, and the second target 8° above or below it (Fig 1B). In the in-flight displacement task, the fixation point appeared to the left screen center when the target was on the right, and to the right of screen center when the target was on the left (the absolute position was selected randomly from one of nine locations positioned around -9° or 9°: -1, 0, 1° left or right; -1, 0, 1° above or below). The target appeared 12° to the left or right of the fixation point, and, upon saccade detection, was displaced to one of 13 equiprobable locations (Fig 2B).

### Peters et al. Delusion Inventory

The Peters et al. Delusion Inventory [18,26] is a self-administered questionnaire designed to quantify delusional-like ideation in the general population. We used the 40-question version of the PDI. Each question describes a belief or mental experience, such as “Do you ever feel/think as if …” (e.g. question 1: “Do you ever feel as if you are under the control of some force or power other than yourself?”). Subjects are asked to respond yes or no, and in the case of a positive response, to evaluate other aspects of the belief or experience (how distressing it is, how often they think about it, etc). In the current study, we used the general score based on the number of positive responses, which could range from 0 to 40. After subjects had performed the two tasks in the laboratory, we emailed them a link to an electronic version of the questionnaire. Subjects identified themselves by a predetermined subject number, which was matched to the behavioral data, such that the questionnaires were filled out and scored anonymously. We used an online version of the questionnaire to ensure anonymity and because we hoped to facilitate spontaneous responses.

### Eye movement recording and analysis

Viewing was binocular. Movements of the right eye were monitored with an Eyelink 1k (SR Research, Mississauga, Ontario, Canada) at 1000 Hz sampling rate. At the beginning of a session, the Eyelink was calibrated with the standard 9-point Eyelink procedure. Before each trial, fixation was checked. If the distance between the fixation check and the calibration was greater than 1.5°, a new calibration was initiated. Calibration was also automatically renewed every 50 trials. On-line saccade detection was based on a boundary criterion: gaze-contingent changes occurred when the eye position crossed one half of the target eccentricity. Eye movement samples were smoothed with the proprietary algorithms provided by SR Research. Instantaneous velocity and acceleration were computed for each data sample and compared to a threshold (30°/sec and 8000°/sec^2^). Saccade onset was defined as two consecutive above-threshold samples for both criteria. Saccade offset was defined as the first sample of a 20-ms period of below-threshold samples.

In the double-step task, on each trial, we identified up to five saccades made after the go-signal to perform the saccade sequence. From this list of saccades, we identified the "best" second saccade, defined as the first saccade occurring that had a vertical component greater than 4° and a horizontal component less than 4°. We then considered the starting position of that saccade as the endpoint of the first saccade, and the endpoint of that saccade as the endpoint of the second saccade, and used these values in the calculation of our correlation.

We did this because when subjects are asked to make a saccade to a single visual target or to a position in space as in the current experiment, they often actually make two saccades: a large primary saccade and a small corrective saccade. The relationship between the error of a primary saccade (i.e. the distance between the primary saccade endpoint and the visual target) and the horizontal component of the corrective saccade is close to 1; it is precisely because the small secondary saccades serve to bring the eyes closer to the target that they are called *corrective*. Our procedure ensured that the second saccade we were considering was a saccade aiming for the second target, and excluded corrective saccades.

Fig 1C presents traces from two subjects. Each line represents one saccade identified with this procedure.

In the in-flight displacement task, on every trial, we identified the first saccade performed after the go-signal, and used that in the analyses.

### Statistical analyses

*Double-step task*: We performed repeated-measures ANOVAs on saccade latency and gain with factors Saccade (First versus Second) and Condition (Memory-guided versus Visually-guided). We measured Pearson correlations between first saccade error and the horizontal component of the second saccade, and between the self-movement index and the PDI score, and tested significance using bootstraps and comparing to the 95% confidence interval. *In-flight displacement task*: Perceptual performance was quantified by fitting cumulative Gaussian psychometric functions to the response data with the maximum likelihood method. Thresholds were defined as the distance, in degrees of visual angle, between the displacement evoking 50% correct responses and the displacement evoking 75% correct, and expressed as Weber fractions by dividing this distance by 10° (the required saccade amplitude). All experiments and analyses were performed with Matlab (version R2014b) using the Psychophysics Toolbox extensions [27–29].

## Results

### Double-step task

Results were collapsed across the four directions (left-up, left-down, right-up, right-down), separately for the visual and memory-guided versions.

Saccade latency was slightly faster in the visually-guided task than in the memory-guided task (first visually-guided saccade: 346±104ms; second visually-guided saccade: 338±132ms; first memory-guided saccade: 366±108ms; second memory-guided saccade: 391±111ms, mean±standard deviation). A repeated-measures ANOVA confirmed this difference (F(1,40) = 8.5, p<0.01). The difference between first and second saccade latency was not significant (F<1). There were very few errors in the order of saccades, in either task (<2% of trials).

**Fig 3:**
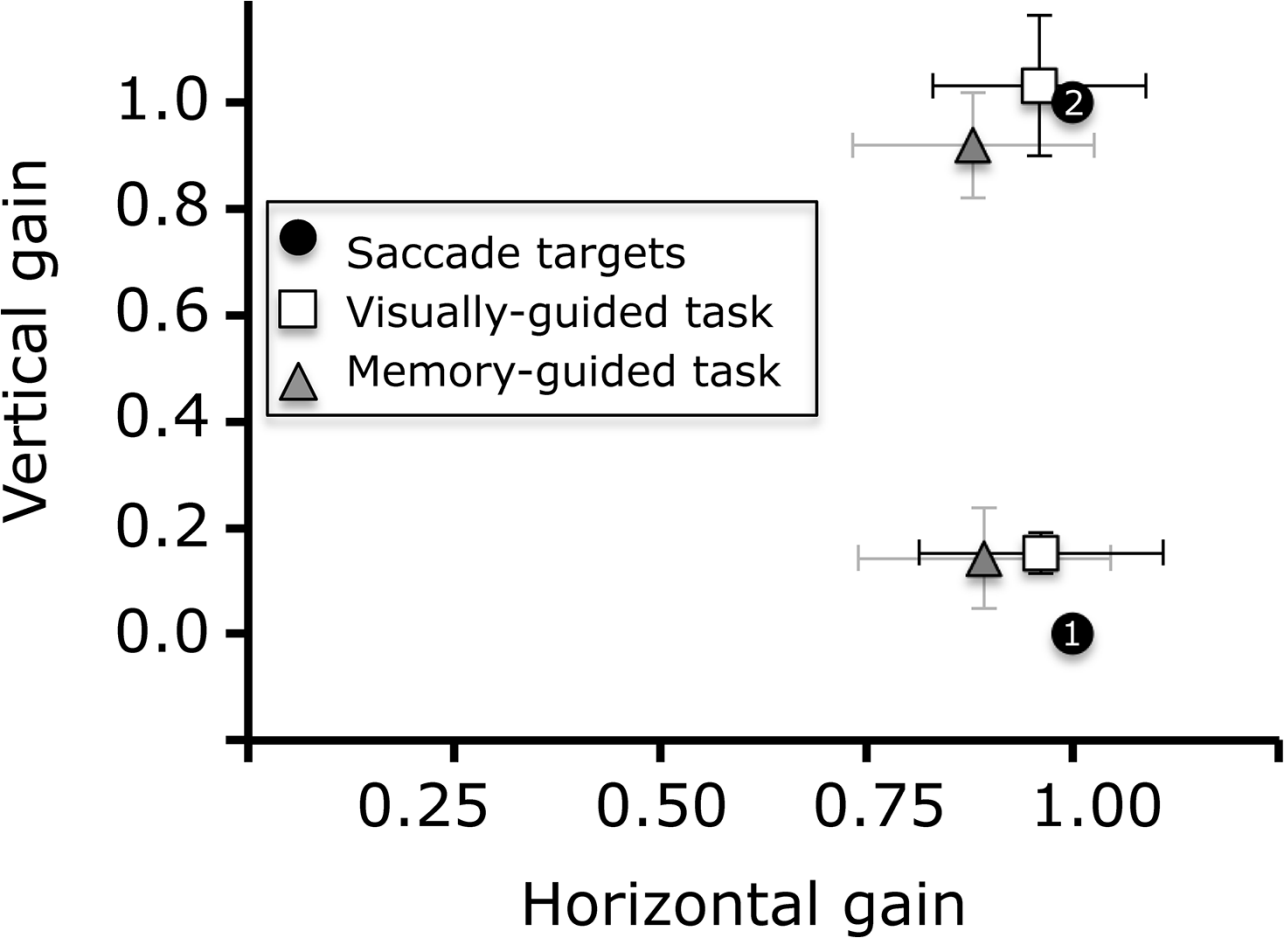
Saccade gain in the double-step task. Vertical and horizontal endpoints of first and second saccades in the double-step tasks.

Saccade gain (saccade amplitude / target eccentricity) was more accurate in the visually-guided task than in the memory-guided task, both for first saccades (0.95±0.15° versus 0.87±0.20°, respectively) and second saccades (0.97±0.17° versus 0.78±0.17°, respectively). The overall difference between visually- and memory-guided tasks was significant (F(1,40) = 21.6, p<0.001). In the visually-guided task, first and second saccade gains were comparable, whereas in the memory-guided task, second saccade gain was smaller than first saccade gain (F(1,40) = 12.2, p<0.001). Fig 3 depicts the saccade endpoints for both conditions.

We had expected that second saccade endpoint in the memory-guided task to be more eccentric than in the visually-guided task, due to a potentially less accurate CD signal relative to the visual signal[30,31]. We found no evidence for this at the group level, as obvious from Fig 3.

**Fig 4:**
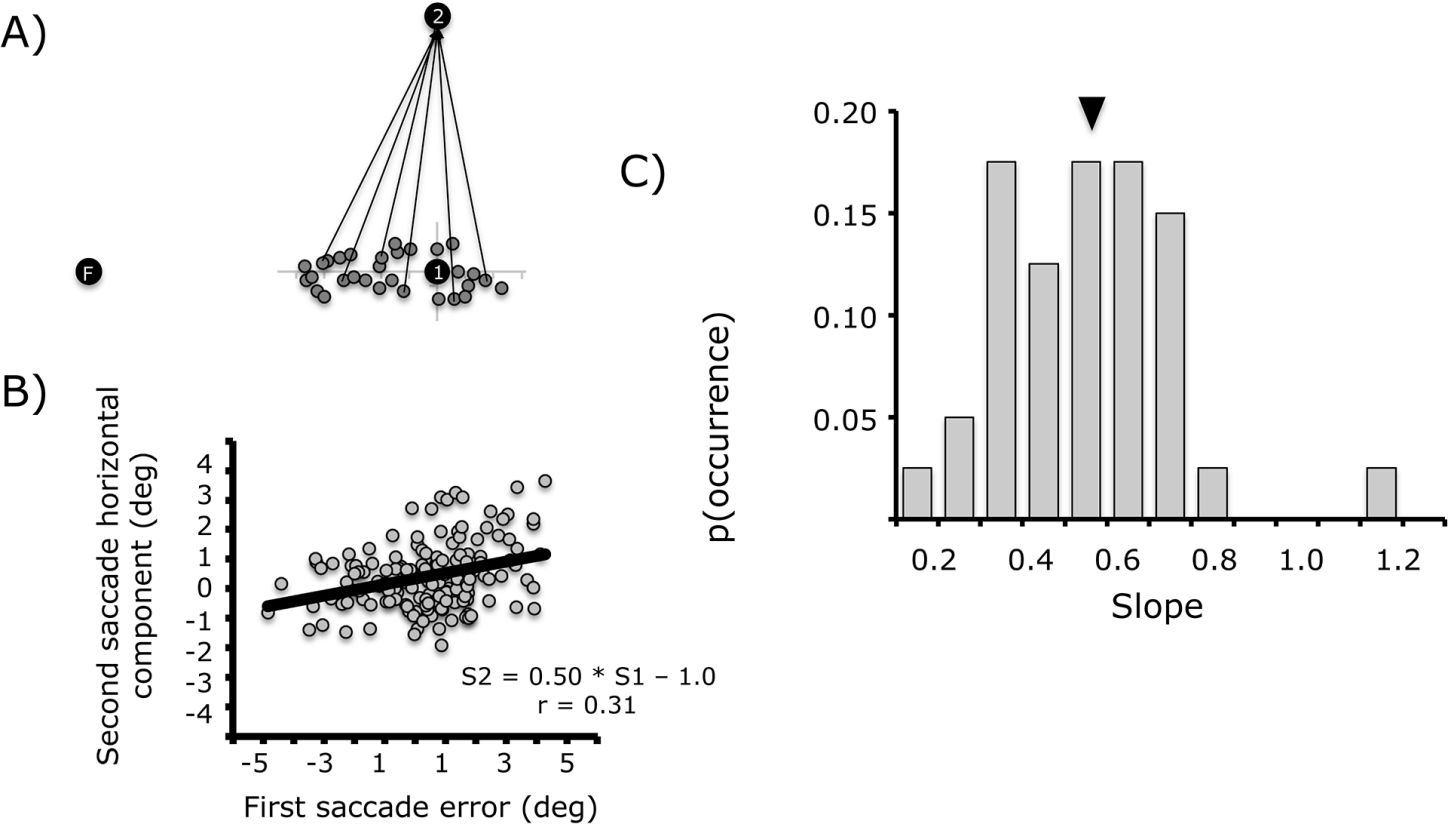
(A) Schematic illustration of first saccade endpoints (grey circles) and a few of the oblique second saccades needed to reach the second target. This schema omits second saccade variability to illustrate the idea of a correlation between first saccade error and second saccade horizontal component. (B) Individual correlation between first saccade error and second saccade horizontal component. (C) Distribution of the slope of the relationship between first saccade error and second saccade horizontal component for the population of subjects. The arrow corresponds to the mean value (0.87°).

To measure the extent to which information about the first saccade was taken into account to plan the second saccade, we examined the relationship between the error on the first saccade and the obliqueness of the second saccade, in the memory-guided task. Indeed, if the first saccade undershot the first target location, then to acquire the second target location, the second saccade should be oblique, as illustrated in Fig 4A. Overshooting saccades should also lead to oblique second saccades but with a horizontal component in the opposite direction. The slope of the relationship between first saccade error and second saccade horizontal component is thus an index of the extent to which the metrics of the first saccade are corrected for in the second saccade. The strength (correlation coefficient) is an index of the precision of that correction. We fit the data with a linear relationship that allows for variance in both x and y.

Overall, we observed a mean correlation of r = 0.18 (bootstrap, 95% confidence intervals = [0.13–0.22]). The mean slope was 0.52 (95% CI = [0.42–0.64]). An individual example and the distribution of observed slopes across our population are illustrated in Fig 4B and 4C. Since the slope is a measure of the extent to which first saccade errors are compensated for by the second saccade, we took slope as our self-movement index.

**Fig 5:**
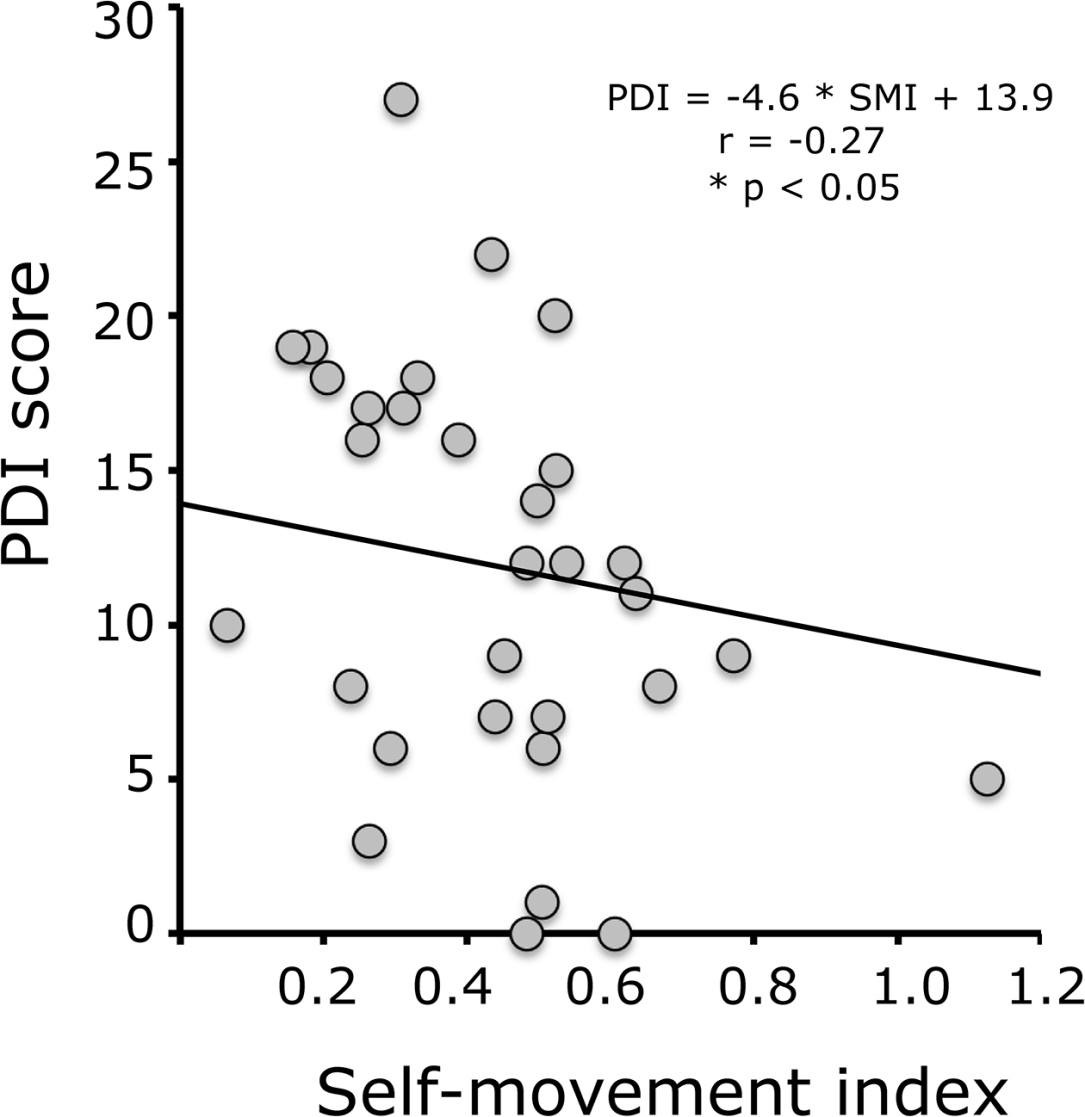
Correlation between the PDI score and the self-movement index in the double-step task.

We then correlated this self-movement index with the PDI score for each participant. The mean score was 11.6 with a range spanning 0 to 27, compatible with previous reports of PDI scores in the general population [17,18,26]. Only 34 of our 41 participants actually completed the online questionnaire, hence the number of points in the scatter plot. There was a significant negative correlation between the self-movement index and the PDI score (bootstrap, r = -0.27, CI = [-0.49 –-0.05]): the greater the self-movement index, the smaller the PDI score (Fig 5).

The correlation was not driven by basic saccade characteristics such as amplitude, variability or latency, as none of these correlated individually with the PDI score (all p>0.05).

### In-flight displacement task

Results were collapsed across saccade directions (left and right). Three participants were unable to perform the task (flat psychometric functions) and were removed from further analysis.

**Fig 6:**
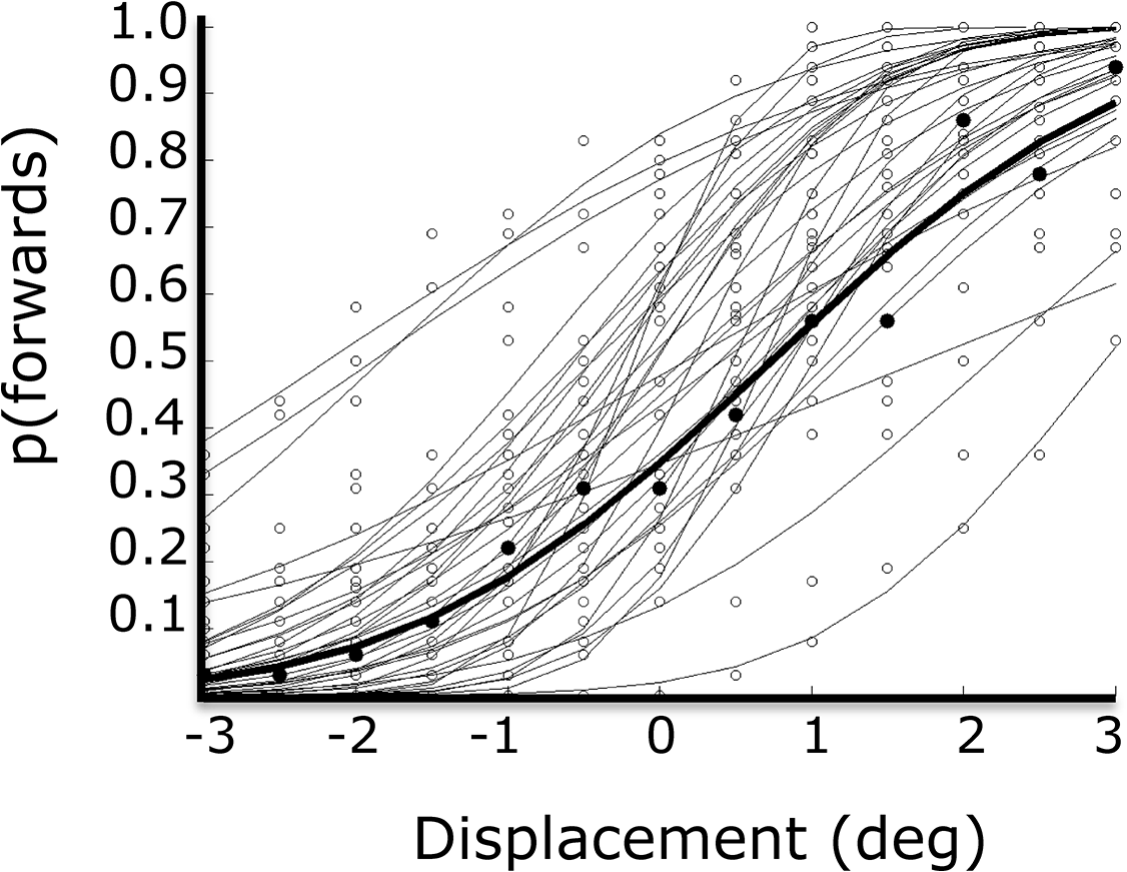
Perceptual performance in the in-flight displacement task. Light grey: individual subjects, thick black: mean.

Saccade latency and gain were normal for visually-guided saccades (196±74 ms; 10.1±1.5°). Average discrimination threshold was 0.10 with a range from 0.04 to 0.27 (Fig 6).

**Fig 7:**
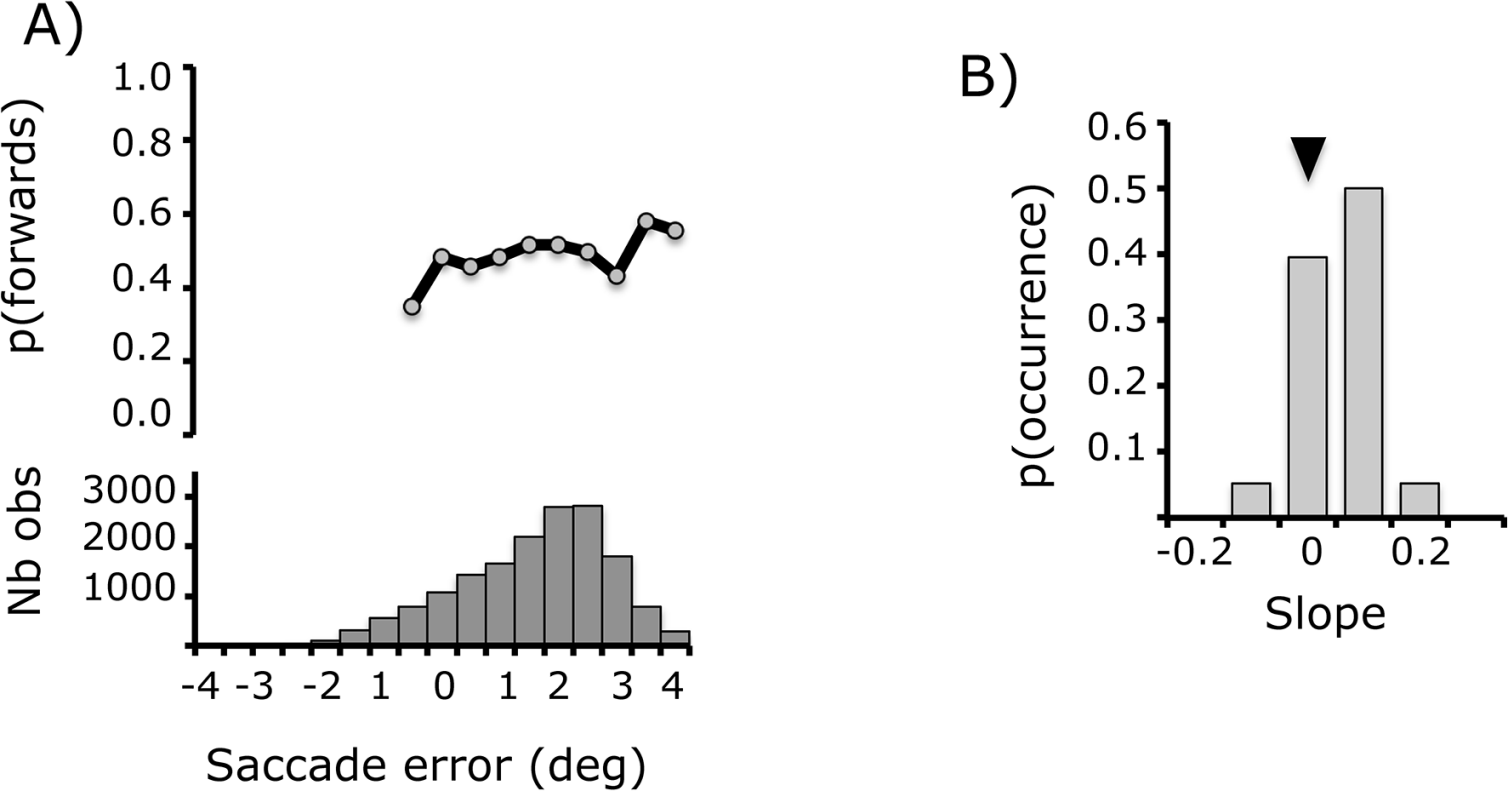
(A) Top panel: Perceptual performance as a function of saccade error, the distance between saccadic endpoint and post-saccadic visual target, with actual displacements subtracted out. Bottom panel: number of observations per error window, calculated over all subjects. (B) Distribution of the slope of the regression between perceptual performance and saccade error for the population of subjects. The arrow corresponds to the mean value (0.07).

Of interest here was the extent to which knowledge about the saccade was revealed in the perceptual responses. For every trial, we calculated the offset between the saccade endpoint and the post-saccadic visual target, and binned trials into windows of 0.5°. For each window, we calculated the proportion of “forwards” responses (Fig 7A). Overall, responses did not depend on saccade endpoint (bootstrap, mean r = 0.07; 95% CI = [-0.14–0.22]). The mean slope was 0.007 (95% CI = [-0.02–0.03]), but there was quite some variation across the population (Fig 7B). The slope constitutes an index of the tendency to attribute self-generated saccade errors to movements of the target (i.e. for every degree of saccade error, the proportion of forwards responses increases).

As for the previous task, we correlated this self-movement index with the PDI. There was no significant correlation (bootstrap, r = -0.07; 95% CI = [-0.39–0.26]).

### Comparison between tasks

We wanted to examine the relationship between self-movement knowledge in the double-step task and self-movement knowledge in the in-flight displacement task. We first looked at the correlation between the self-movement indices obtained in the two tasks: it was non-significant (bootstrap, r = 0.04, 95% CI = [-0.27–0.38]). We then examined to what extent the distributions of self-movement indices in the double-step versus in-flight displacement tasks were different with a Kolmogorov-Smirnov test. The result showed that the two distributions were significantly different (p = 0.00001).

## Discussion

Forty-one healthy adults performed two classic visuo-motor tasks. The double-step task reveals the extent to which knowledge about just-performed saccades, in the absence of visual reafference, is available for preparing subsequent saccades [19]. The presence of such extra-retinal information during the performance of saccade sequences is thought to reveal the use of a corollary discharge (CD) signal for motor planning. Replicating previous results, our subjects’ performance revealed the use of such CD signals [32,33,21,20]. The advantage of the double-step task is to provide a quantitative measure of the degree to which the CD signal is taken into account in planning saccade sequences. This self-movement index correlated, in our participants, with a questionnaire assessing the tendency for delusional-like thinking in healthy subjects: individuals with higher scores showed poorer self-movement information use.

Impaired performance in the double-step task may reflect a faulty self-movement signal, or inappropriate use of this signal by the motor system; our data do not allow us to dissociate these two options.

The in-flight displacement task investigates how the trans-saccadic correspondence of retinal locations is achieved [22,23,25,34]. The extent to which perceptual responses depend on saccade metrics is a quantitative index of the degree to which a prediction about the sensory consequences of the saccade is used for trans-saccadic correspondence. This self-movement index did not correlate, in the same participants, with the delusional-like thinking score.

Although corollary discharge *may* be used in both the double-step and the in-flight displacement tasks, it is not certain that it always *is*. Indeed, observers may use exocentric cues such as screen borders to keep track of the locations of targets. Several arguments point away from the preponderant use of exocentric cues in the current experimental conditions (other than the fact that the room was dark). The influence of exocentric cues in the in-flight displacement task has been shown to be minimal at the distance that the screen borders were in the current experiment [35]. Observations from our own laboratory confirm the absence of an effect of screen borders on performance in this task (Wexler & Collins, unpublished observations). Using exocentric cues in the double-step task would lead to an absence of correlation between first and second saccade errors, which is not what we found.

Several previous studies suggested that the mechanisms responsible for sensory suppression during self-generated movements, which likely depend on an accurate CD signal, are altered in schizophrenic patients. Shergill and colleagues [10] asked healthy adults to match the force of a tactile stimulus delivered to one index finger by pressing with the opposite index finger. The force produced by participants was systematically stronger than the initially delivered stimulus, suggesting an underestimation of the tactile sensation produced by an active finger press relative to a passively-sensed stimulus. Such an underestimation suggests that a prediction about the sensory consequences of one’s own finger press attenuate the perception of tactile pressure, and indeed cerebral correlates of suppression in primary tactile areas has been reported [36]. Schizophrenic patients, however, performed almost perfectly in this task, and showed lesser cerebral suppression, as expected if no prediction about the sensory consequences of their own finger press occurred [10,12]. Ford and colleagues [7,8,37,38] obtained similar results in the auditory modality. Moreover, deficits in self-movement information use are observed mostly in schizophrenic patients presenting deficits in attribution of agency [39,40,13,41]. For example, in a task that required cancelling out self-induced retinal movement to accurately perceive moving dots, no difference between patients and healthy controls was observed. However, there was a correlation between a clinical score of delusions and the ability to compensate for self-induced retinal movement in patients [13].

Our study did not include clinically diagnosed schizophrenic patients, and it is not clear how our non-clinical population relates, functionally and cerebrally, to clinically diagnosed schizophrenics. It is therefore difficult to directly relate our findings to the studies described above. However, testing a non-clinical population avoids confounding factors of illness history and neuroleptic medication. By exploiting individual differences in sensory prediction accuracy in the general population, and correlating it with a measure of delusional-like thinking, we tested the sensory-prediction model of delusions [6] without such confounding factors. To our knowledge, only one other study has measured delusional-like ideation in healthy subjects and related that to performance in a task requiring CD information. Teufel et al. [17] asked healthy subjects to perform the force-matching task described above, and to respond to questionnaires assessing schizotypy and delusional-like thinking (including the PDI). Their results revealed a correlation between the degree of sensory attenuation and the PDI score: subjects with higher levels of delusional thinking showed more accurate force matching (i.e. less sensory attenuation). These results provide experimental evidence supporting the view that not only delusions of influence in schizophrenia, but also delusional-like thinking in healthy subjects, might be related to a specific deficit of the sensory-predictive system [17]. Deficits in predictive activity may predispose towards the emergence of these symptoms under attenuated, non-pathological forms of reasoning and thinking.

Our results fit well with the Teufel et al. [17] study, since subjects with higher levels of delusional thinking showed less CD use in saccade planning. There was no such correlation, however, with the degree of CD use in trans-saccadic visual perception. A similarity between the double-step task and the force-matching task is that they both involve CD mechanisms in a motor task, whereas the in-flight displacement task involves CD for visual perception. In this sense, our results also fit well with the studies cited above in which deficits in motor tasks (force-matching, speech) were observed in patients. They might be at odds, however, with the study showing a correlation between a clinical score of delusions and the ability to compensate for self-induced retinal movement [13], since their task is similar to our in-flight displacement task in that it requires correcting for eye movements in visual perception. It is difficult, however, to directly compare these results because of differences in methods and of tested populations.

We propose that the differential correlation with the PDI scores, and the absence of a correlation between the two self-movement indices, reveals a functional dissociation between CD-for-perception and CD-for-action. In the double-step task, a CD serves subsequent action. In the in-flight displacement task, CD serves visual perception. Although our results are behavioral, they may also point to distinct cerebral pathways that carry CD signals either for motor planning or for trans-saccadic correspondence.

One cerebral pathway carrying corollary discharge has been identified in the macaque monkey [30] and the brain damaged human patient [31]. It leads from the superior colliculus to the frontal eye fields via mediodorsal thalamus, and carries metric information about a given saccade that reaches the frontal eye fields before its execution. Visual neurons in the frontal eye fields differentiate retinal changes due to saccades as opposed to object displacements [42], suggesting that this pathway might be crucial for trans-saccadic correspondence. Inactivation of medio-dorsal thalamus in the macaque and its lesion in the human patient lead to small but systematic deficits in the double-step task [43,31]. In the brain-damaged patient, perceptual deficits in the in-flight displacement task are much larger, and suggest that the colliculo-frontal pathway is critically involved in trans-saccadic perceptual correspondence [24,31].

Double-step task deficits such as those expected with a faulty CD signal have been reported in brain-damaged patients. Central thalamic lesions of the internal medullary lamina, but sparing the mediodorsal thalamus, lead to such deficits [44]. Furthermore, patients with frontal damage did not present CD deficits in the double-step task, but patients with parietal damage did [45,46]. We suggest that the parietal cortex, possibly via a colliculo-parietal pathway, is critical for CD use in saccade sequence planning. We propose that there are two distinct brain pathways for CD, a frontal pathway serving perception and a parietal pathway serving action. The frontal pathway would mediate perceptual judgments and thus may relate to visual awareness. According to this view, our results may point to a selective involvement of the parietal pathway in schizotypical traits (as measured by the PDI).

The neuropathology of schizophrenia has been extensively studied and has revealed reduced brain volume, abnormal functional activity and characteristic changes in microcircuitry in many brain regions, particularly in frontal, parietal and temporal areas[47,48]. No study to date can directly pinpoint a selective deficit of one CD carrying pathway and not another, or even a fronto-parietal dissociation; thus our proposal remains speculative. However, convergent studies of eye movements may suggest such a dissociation between frontal activity and posterior activity in subjects with schizotypal personality. While both patients with schizophrenia and subjects with schizotypal personality share common abnormalities in posterior brain regions (occipital cortex, striatum, thalamus, cerebellum for smooth eye movements and posterior intra parietal sulcus for prosaccades), frontal activity is specifically reduced in schizophrenia but not in schizotypy [49–51].

Our results fit with current attempts to understand psychiatric disorders, in particular schizophrenia, within the framework of the brain as a predictive machine[6,52,53]. According to this view, the brain compares top-down predictions and bottom-up prediction errors to infer the causes of sensory information. In this framework, schizophrenia can be seen as false inference: attribution of sensory information to an incorrect source (self versus other) may lead to delusions and hallucinations. Our results suggest that the quality of false inferences may depend on whether the task requires perceptual judgments or motor acts.
